# The Meldrum Forced-Draught Furnace

**Published:** 1921-03-12

**Authors:** 


### The Meldrum Forced-draught Furnace.

The Meldrum furnace and the Meldrum mechanical stoker have been a long time on the market, and have done very good work. There are two special features in connection with the furnace: The firebars, which correspond to the bars of the ordinary domestic grate, are placed very close together, so that very inferior fuel and dust fuel can be employed. The bars have been specially designed to allow sufficient air to pass up between them, so that combustion of the fuel can go on freely in the furnace; but the fuel itself has very little chance of falling through between the bars, so that there is no waste. It will be understood that the use of low-grade fuel, the dust that is sold at a very low price, providing that it can all be burnt, and burnt satisfactorily, leads to economy in fuel costs. The other important feature of the Meldrum furnace is the draught is created by means of steam jets placed under the firebars, the jets creating a lowered pressure in their neighbourhood, and the air from the atmosphere flowing in through passages provided for it to the firebars, and up through the bars in the usual way. The steam for the jets is superheated and deprived of all moisture carried in suspension before it is allowed to enter the pipes leading to the jets. It is claimed that in this way moisture is prevented from reaching the fuel, and that by the use of the steam jets clinkers are prevented from adhering to the firebars, and the furnace grates are therefore more easily cleaned. The fine ash that is formed by the burning of the coal falls easily through the spaces between the bars, leaving them free for the passage of the air. The thinness of the bars and the cooling action of the steam and air are stated to give the bars a long life.

